# Supplementary material for: The Anticancer Ruthenium Compound BOLD-100 Targets Glycolysis and Generates a Metabolic Vulnerability towards Glucose Deprivation
Source: Pharmaceutics. 2022 Jan 20;14(2):238. doi: 10.3390/pharmaceutics14020238 (PMC8875291; doi:10.3390/pharmaceutics14020238)
Supplement: Supplementary file 1 [file pharmaceutics-14-00238-s001.zip › pharmaceutics-1546757-supplementary.pdf]

pharmaceutics

MDPI

Supplementary Materials: The Anticancer Ruthenium Compound BOLD-100 Targets Glycolysis and Generates a Metabolic Vulnerability towards Glucose Deprivation

Dina Baier, Beatrix Schoenhacker-Alte, Mate Rusz, Christine Pirker, Thomas Mohr, Theresa Mendrina, Dominik Kirchhofer, Samuel M. Meier-Menches, Katharina Hohenwallner, Martin Schaier, Evelyn Rampler, Gunda Koellensperger, Petra Heffeter, Bernhard Keppler and Walter Berger

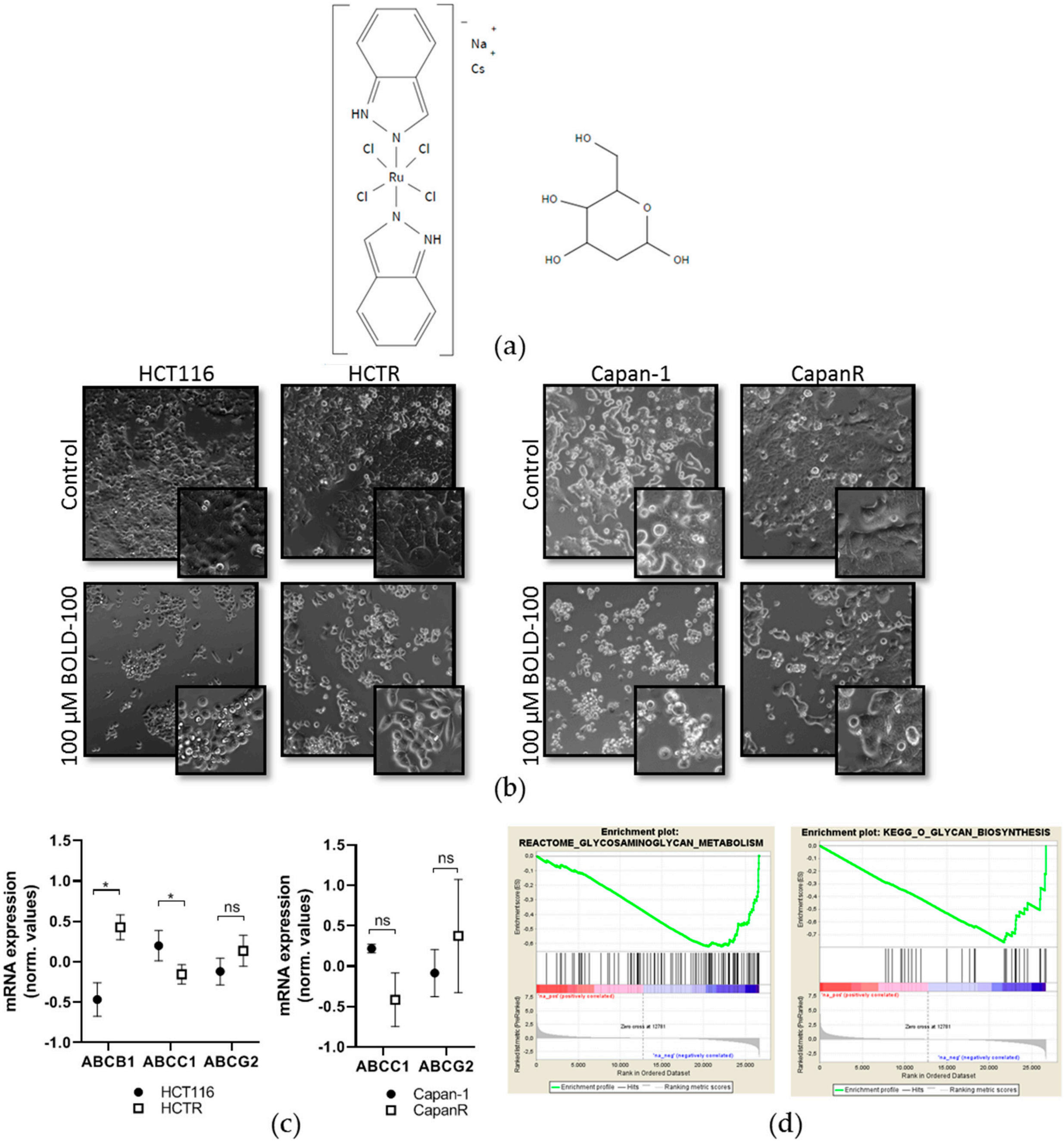

Figure S1 BOLD-100 treatment affects the cellular carbohydrate metabolism and acquired resistance is independent from drug transporter expression changes (**a**) Chemical structures of BOLD-100 (left) with cesium as an intermediate salt form and 2-DG (right)

created with SciFinder (Copyright © 2022 American Chemical Society). (**b**) Representative phase-contrast photomicrographs (4x magnification) after 24 h of treatment with 100 µM of BOLD-100 to indicate morphologic changes of parental and acquired BOLD-100-resistant HCT116 cells. Enlargements are 10x magnified. (**c**) Normalized mRNA expression of ABC drug transporters ABCB1, ABCC1, and ABCG2 in HCT116 and HCTR or Capan-1 and CapanR cells. In Capan cell models, ABCB1 mRNA expression was below the detection limit. The statistical significance of differences was calculated with an unpaired two-tailed Student´s t-test: *$p<0.05$; ns: non-significant. (**d**) GSEA identified "GLYCOSAMINOGLYCAN_METABOLISM" (nominal *p*-value < E^{-7}, FDR q-value = 0.003, and normalized enrichment score (NES) = -1.861) as top fourth downregulated gene set according to the REACTOME database and "O_GLYCAN_BIOSYNTHESIS" (nominal *p*-value < E^{-7}, FDR q-value = 0.003, and normalized enrichment score (NES) = -1.847) as top seventh downregulated gene set according to the KEGG database upon 100 µM BOLD-100 treatment for 6 h in HCT116 cells.

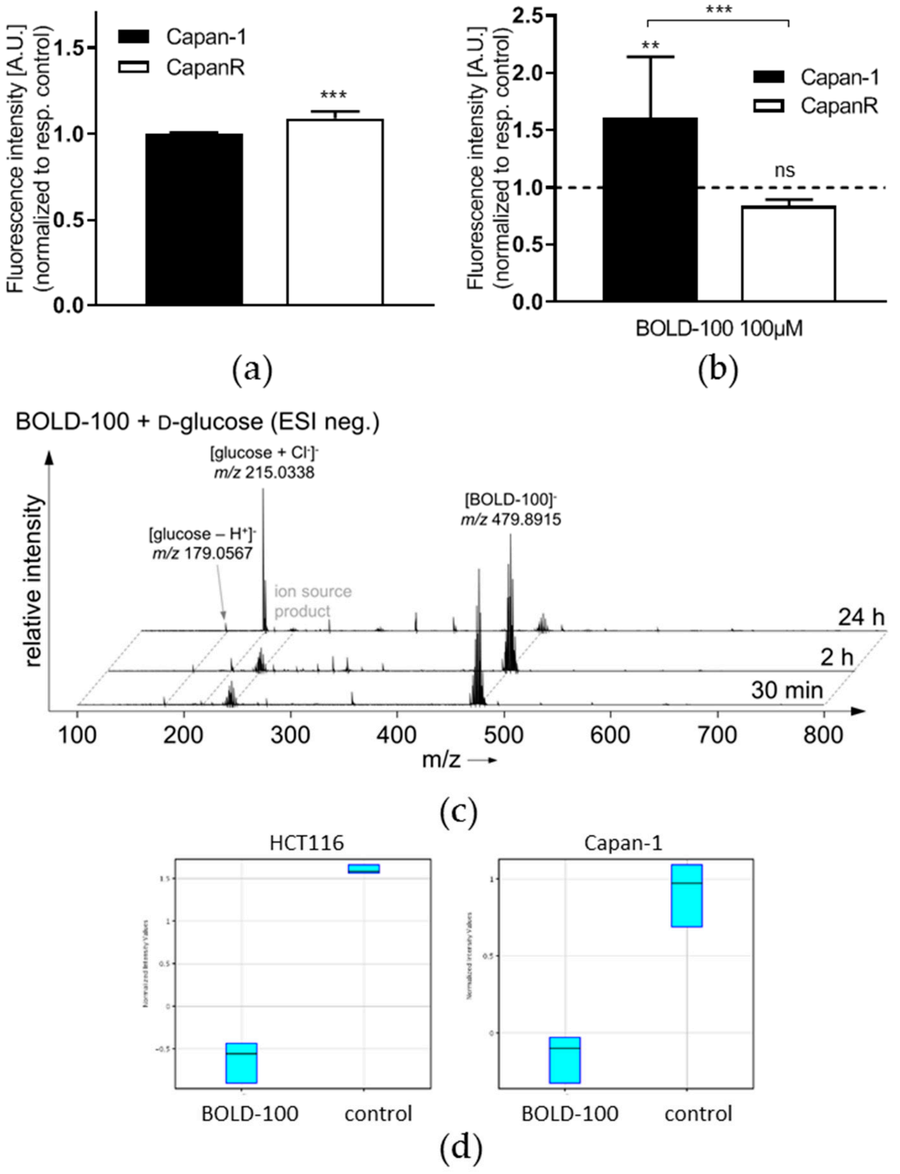

Figure S2 Acquired BOLD-100-resistance is characterized by enhanced glucose uptake and BOLD-100 treatment reduces HK2 expression independent from drug-glucose adduct formation. FACS analysis of fluorescence intensity (arbitrary units, a.u.) of glucose-starved Capan-1 and CapanR cells after 1 h incubation with 25 µM of 2-NBDG following treatment with solvent control (**a**) or 100 µM of BOLD-100 (**b**) for 24 h. Results are mean of triplicates ± SD of two independent experiments. In (**b**) data are normalized

to the respective control (dashed line). Statistical significance of differences was calculated with a two-tailed unpaired student´s t-test (**a**) or two-way ANOVA with Tukey´s multiple comparisons test (**b**): ***p<0.001; ns: non-significant**.** (**c**) Mass spectra of the interaction between BOLD-100 and D-glucose (1 : 4 molar ratio) acquired in the negative ion mode after the indicated incubation times. (**d**) Relative mRNA expression of HK2 in HCT116 and Capan-1 cells with or without 6 h treatment with 100 µM of BOLD-100.

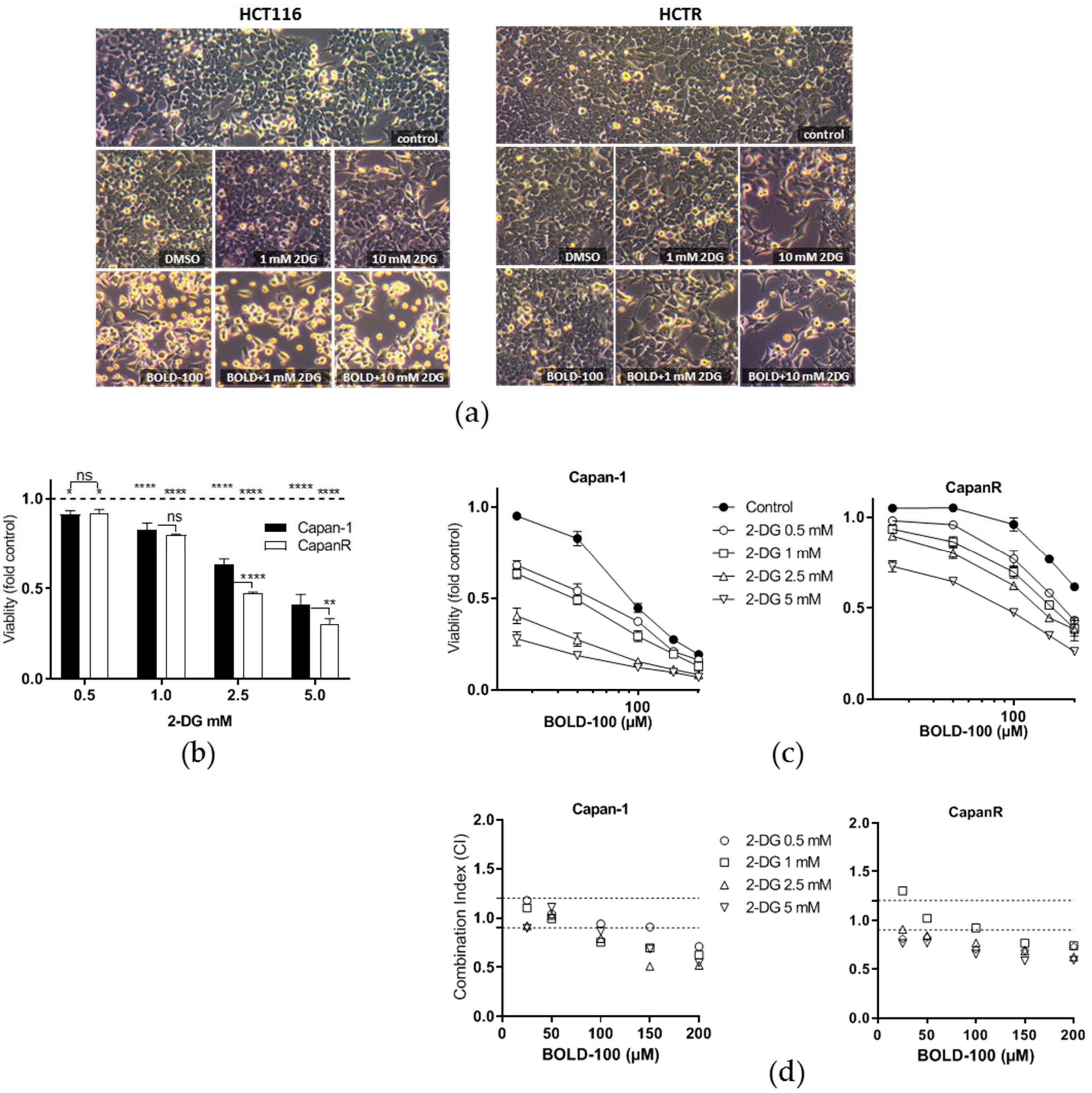

Figure S3 Acquired BOLD-100-resistant Capan-1 and HCT116 cells are hypersensitive to 2-DG and the anticancer effect can be boosted in combination with BOLD-100. (**a**) Respective phase-contrast photomicrographs (10x magnification) of HCT116 and HCTR cells after 24 h of treatment with 100 µM of BOLD-100 or the indicated concentrations of 2-DG, or the combination of both compounds to indicate morphologic changes. Two different control states were included, i.e. the medium control without and with DMSO. (**b**) Cell viability assay of Capan-1 vs. CapanR cells after 72 h of treatment with 2-DG relative to respective controls (dashed line). The statistical significance of differences was determined using a two-way ANOVA with Tukey´s multiple comparisons test: **p<0.01, ****p<0.0001 (**c**) Cell viability of Capan-1 and CapanR cells upon 72 h treatment with BOLD-100 in combination with 2-DG at the indicated concentrations determined by MTT assay. Data are mean of triplicates ± SD. One representative of three independent experiments is shown. (**d**) Combination indices (CI) based on cell viability data from Capan-1 and CapanR cells treated with BOLD-100 in combination with 2-DG at the indicated concentrations for 72 h. CI < 0.9, synergism; CI = 0.9-1.2, additive effects; or CI > 1.2 antagonism.

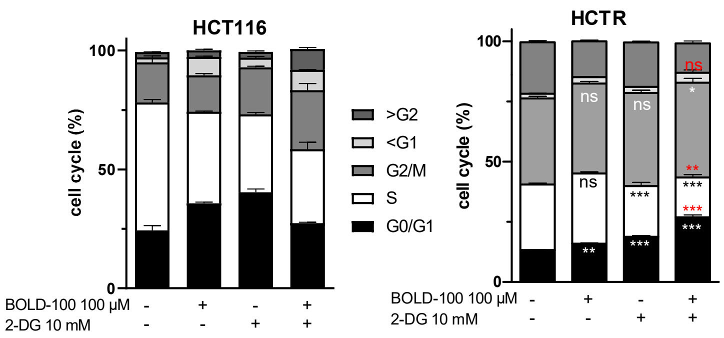

(a)

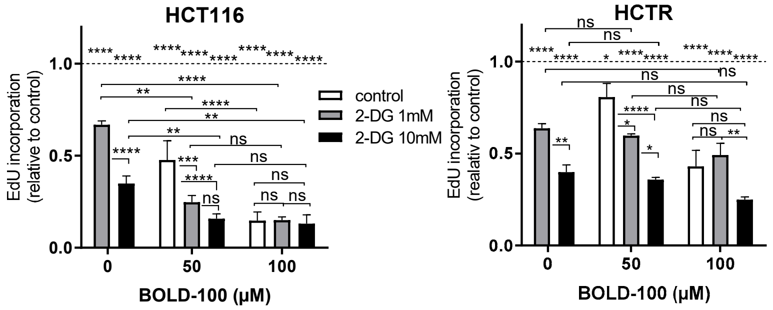

	antiproliferative IC_{50}:	
	HCT116	HCTR
BOLD-100 (µM)	47.95	90.74
2-DG (mM)	5.76	6.20

(b)

Figure S4 Regulation of cell cycle distribution and proliferation by 2-DG and BOLD-100. (**a**) Cell cycle analysis determining the DNA content of the indicated ethanol-fixed, PI-stained cells after 24 h of treatment with the indicated drug concentrations by flow cytometry. Controls contained the amount of DMSO corresponding to 100 µM of BOLD-100. Percentages of 50.000 cells in G0/G1, S, G2/M, and <G1 or >G2 phase of cell cycle were calculated by FlowJo software. The statistical significance of differences was determined using a one-way ANOVA with Tukey´s multiple comparisons test: *$p<0.05$, **$p<0.01$, ***$p<0.001$; ns: non-significant. Black and white asterisks indicate significant difference as compared to the control and red asterisks show significant differences compared to the single treatments. (**b**) EdU incorporation assay of HCT116 and HCTR cells after 24 h of treatment with either 2-DG or BOLD-100 or their combination at the indicated concentrations relative to respective controls (dashed line). Controls contained the amount of DMSO corresponding to 100 µM of BOLD-100. Data are mean of triplicates ± SD of one representative experiment. The statistical significance of differences was determined using a two-way ANOVA with Tukey´s multiple comparisons test: *$p<0.05$, **$p<0.01$, ***$p<0.001$, ****$p<0.0001$; ns: non-significant. Respective significance levels relative to the controls are indicated above the dashed lines. Significance between respective treatment groups is given beneath the dashed line. The table gives IC_{50} values of the drug-respective antiproliferative activity.

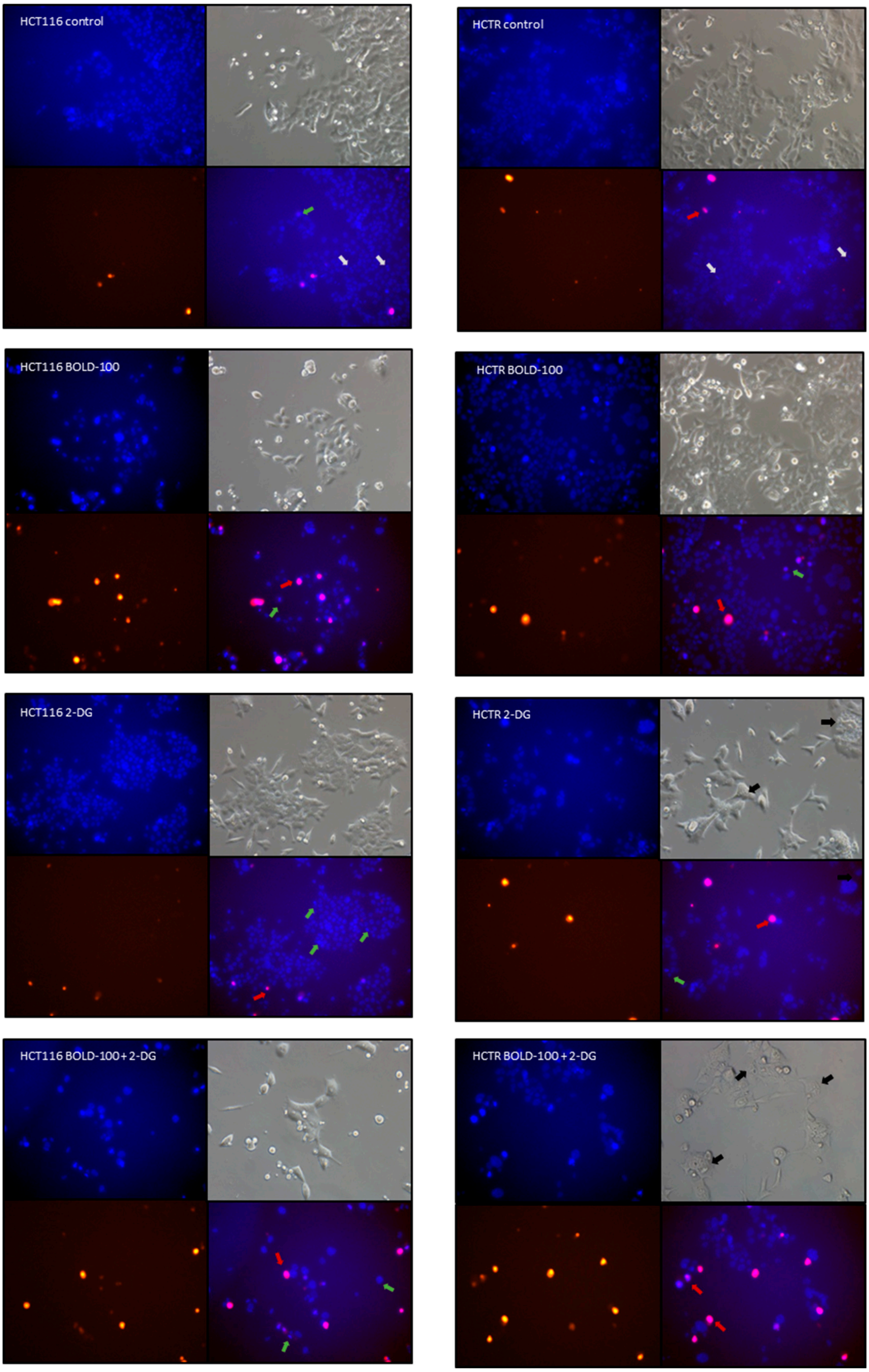

Figure S5 Morphological assessment of apoptosis induction under 2-DG and BOLD-100 treatment by Hoechst 33342/PI dual-staining. Representative images of Hoechst 33342 (blue)/PI (red)-stained HCT116 or HCTR cells after treatment for 72 h with DMSO (control, equivalent to BOLD-100), 2.5 mM of 2-DG or 100 µM of BOLD-100 or their combination. Images were taken by fluorescence microscopy (magnification 20x). Phase contrast images display corresponding cellular morphological changes. Black arrows exemplarily indicate distinct morphological changes induced by 2-DG treatment.

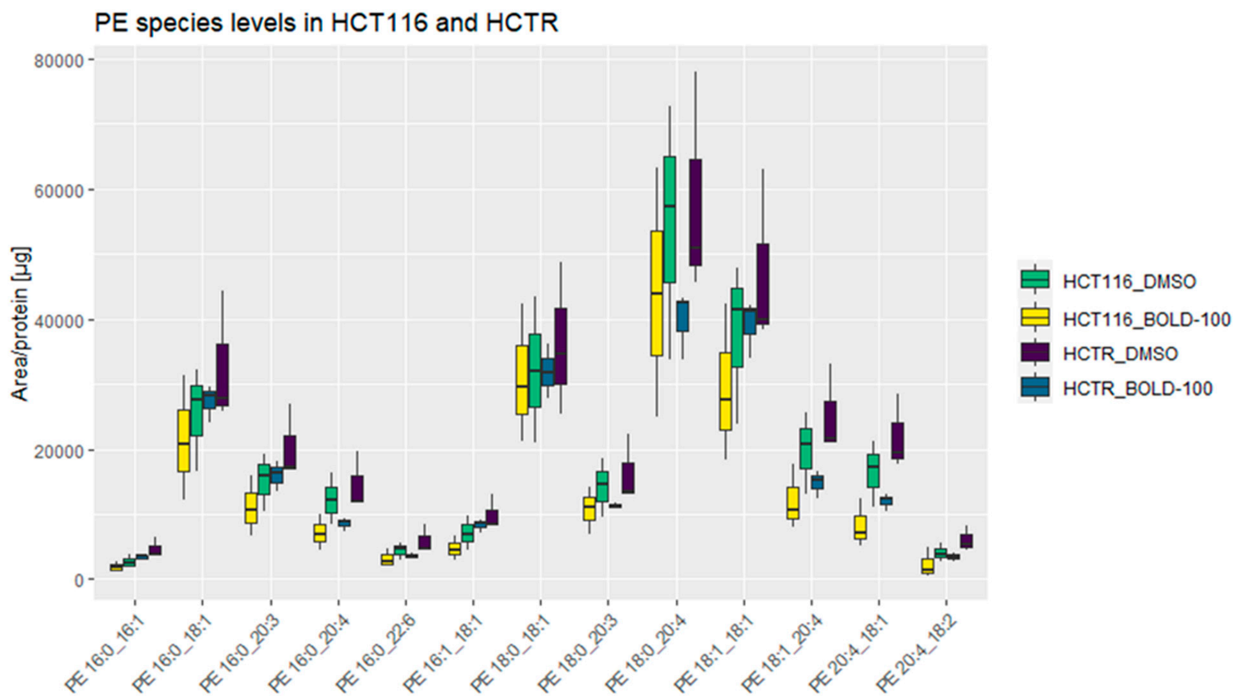

Figure S6 Regulation of cellular phosphatidylethanolamine levels by BOLD-100. Lipidomics analysis of HCT116 and HCTR cells treated with 100 µM of BOLD-100 or the corresponding amount of DMSO for 24 h. Samples were analyzed with RP-UHPLC-HRMS followed by data evaluation in LipidSearch 5.0 and R Studio. All groups were measured in triplicates, integrated areas were normalized to the protein content [µg].

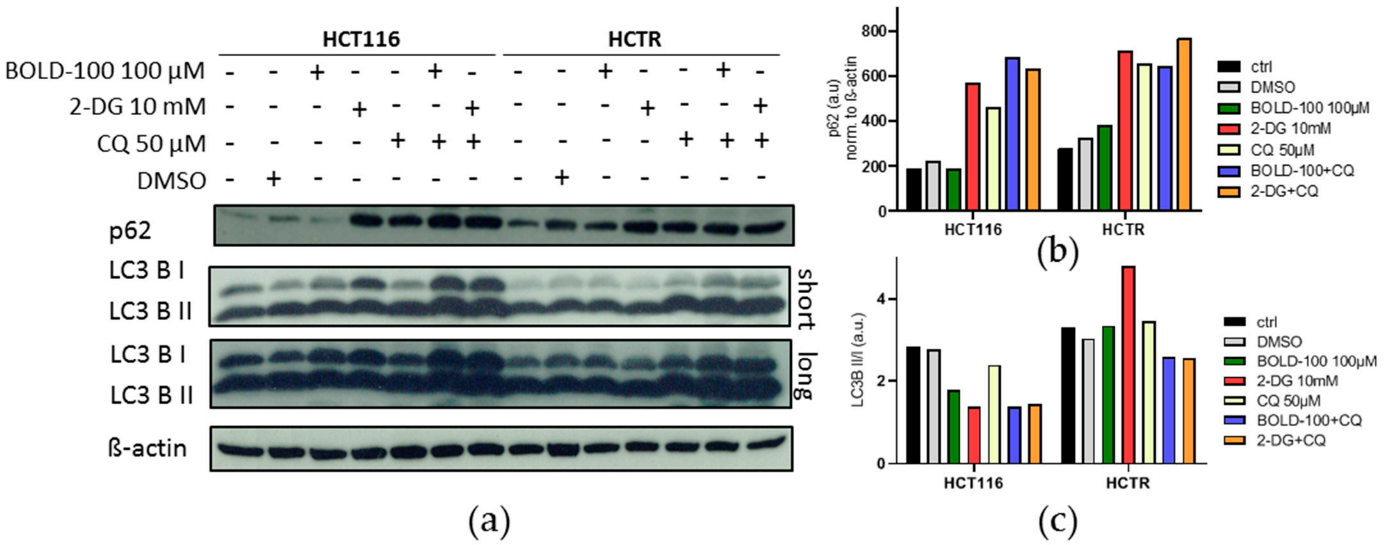

Figure S7 Autophagy regulation by BOLD-100 and 2-DG under autophagic flux blockade by CQ. (**a**) Expression levels of p62, and LC3B I/II in HCT116 and HCTR cells treated with 100 µM of BOLD-100, 10 mM of 2-DG, 50 µM of CQ, or their combination for 24 h analyzed by Western blotting. Two different control states were included, i.e. medium control for 2-DG and CQ or DMSO as solvent control for BOLD-100. β-actin served as loading control. LC3B signals are depicted with shorter (upper) and longer (lower) exposure times. (**b**) Quantification of treatment-respective p62 intensities of Western blot signals depicted in (a). (**c**) Calculation of treatment-respective LC3B II/I ratio from protein expression detected in (a).
